# Traditional Chinese medicine Ka-Sai-Ping suppresses the growths of gastric cancers via induction of autophagy

**DOI:** 10.18632/oncotarget.18041

**Published:** 2017-05-20

**Authors:** Mo-Li Zhu, Jun-Xiu Lu, Guo-Pin Pan, Song Ping, Fan-Rong Zhao, Heng-Tian Qi, Hai-Ya Yu, Xu Jian, Guang-Rui Wan, Peng Li

**Affiliations:** ^1^ College of Pharmacy, Xinxiang Medical University, Xinxiang, China; ^2^ Department of Histology and Embryology, Xinxiang Medical University, Xinxiang, China; ^3^ San-Quan College of Xinxiang Medical University, Xinxiang, China; ^4^ Department of Cardiothoracic Surgery, The Third Affiliated Hospital, Xinxiang Medical University, Xinxiang, China; ^5^ Department of Neurology, The People's Hospital of Xishui County, Huangang, Hubei, China

**Keywords:** traditional Chinese medicine, Ka-Sai-Ping, gastric cancer, autophagy, apoptosis

## Abstract

Traditional Chinese medication is increasingly used to treat a wide range of human chronic diseases like cardiovascular diseases and cancers. This study was designed to explore whether ka-sai-ping (KSP), a novel traditional Chinese medicine developed by us, prevents gastric cancer growths and to investigate the underlying mechanism. The xenograft model of mouse gastric cancer was established by injecting MFCs into nude mouse subcutaneously. Cell autophagy was assessed by MDC staining. Lysosome and mitochondria were detected by Lyso-Tracker Red and Mito-Traker Green staining. Incubation of cultured mouse gastric cancer cell line MFCs with KSP for 48 hours, concentration-dependently reduced cell survivals and activated autophagy, which were accompanied with damaged lysosomes and mitochondria. *In vivo* studies indicated that KSP therapy (20 ml/kg/day) for two weeks suppressed the growth of gastric cancer, increased the protein levels of LC3-II, beclin-1, cathepsin L, bcl-2, p53, and capase-3 in tumor tissues from the xenograft model of mouse gastric cancer. Importantly, all these effects induced by KSP were abolished by co-administration of autophagy inhibitor 3-MA. In conclusion, KSP activates cell autophagy to suppress gastric cancer growths. Clinically, KSP is potentially considered as a medicine to treat patients with gastric cancer.

## INTRODUCTION

Gastric cancer or gastric carcinoma is a kind of malignant tumors worldwide [1]. Till now, chemotherapy is the mainstay approach for patients with gastric cancer [2]. However, gastric cancer usually develops resistance to chemotherapeutic drugs with a short life of disease control, and most patients would go to die within one or two years [3, 4]. To combat the mortality rates from gastric adenocarcinoma, some new drugs are in want of.

Traditional Chinese medicine is widely used in China and other countries in East Asia [5, 6]. Typically, formulae are in consist of minerals or herbs, in which one represents the principal component, and others serve as adjuvants to increase the effects of principal component. In some formulae, multiple components could interact multiple targets and produce high efficacies.

The complexity of traditional Chinese medicine demonstrates that treatment protocols are able to be carefully designed to fight a disease. Therefore, we have generated a traditional Chinese compound prescription of xin-mai-jia to treat patients with atherosclerosis in China by improving endothelial function [7]. In this way, we developed a novel traditional Chinese compound prescription of ka-sai-ping (KSP) to prevent gastric cancer in light of the theory of traditional Chinese medicine and the pathophysiological mechanism of gastric cancer.

## RESULTS

### KSP reduces gastric cancer cell survivals

**Figure 1:**
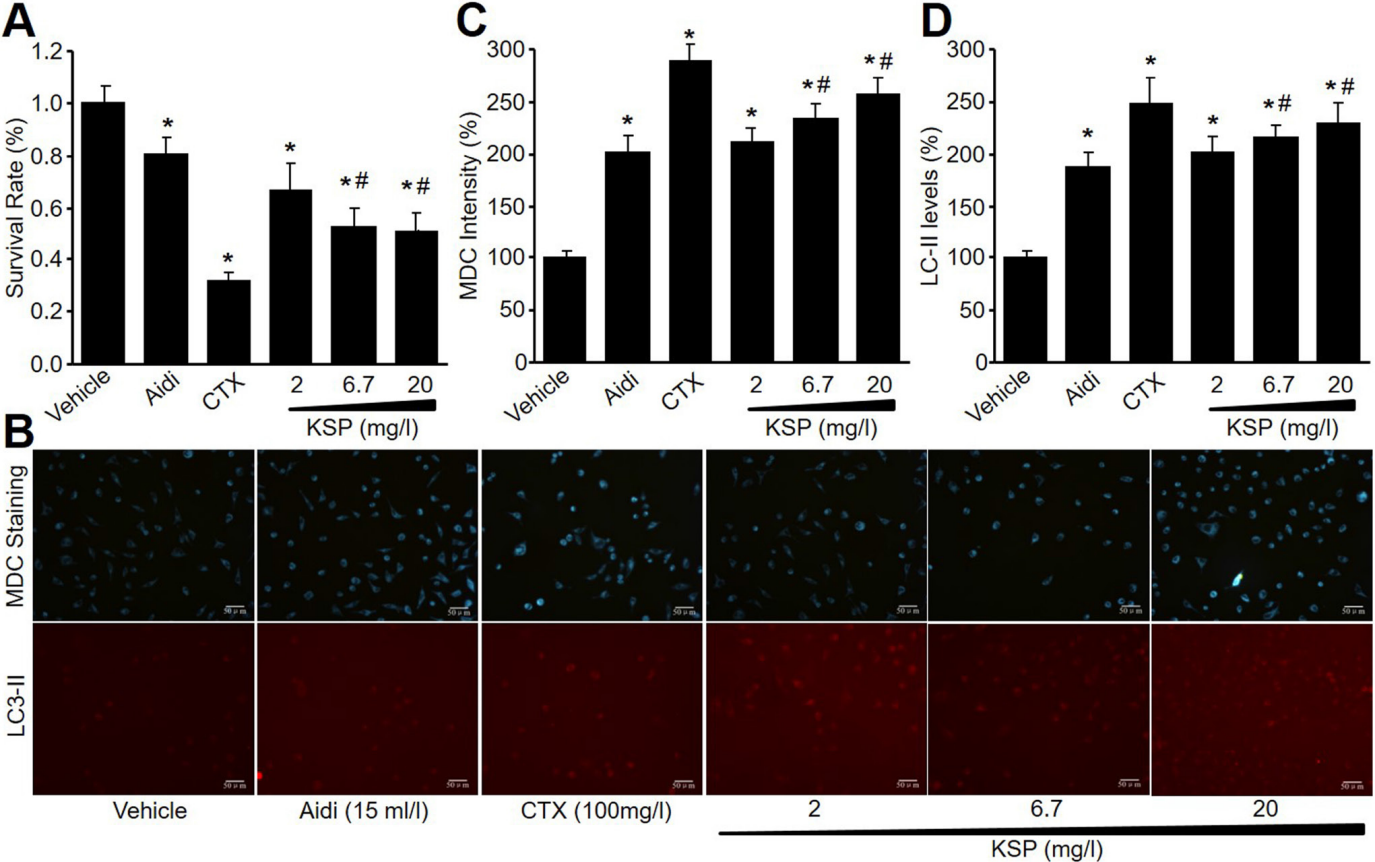
KSP reduces cell survival and increases autophagy in cultured MFCs MFCs were incubated with Aidi (15 ml/l), CTX (100 mg/l), and KSP (2, 6.7, 20 mg/l) for 48 hours. Cell survival was detected by CCK8 assay in (**A**). Autophagy was determined by MDC staining and CY3 staining in (**B**). The quantitative analyses of autophagy in (**C**) and LC3-II in (**D**) were performed in pictures from B. N is 3 in each group. *P < 0.05 *VS* vehicle, ^#^*P* < 0.05 *VS* 2 mg/ml KSP.

To investigate the effects of KSP on gastric cancer cell growth, mouse gastric cancer cell line (MFC) was cultured and treated with KSP (2, 6.7, 20 mg/l) for 48 hours. Cyclophosphamide (CTX, 100 mg/l) and Chinese medicine injection Aidi (15 ml/l), served as positive controls. Cell viabilities were detected by CCK8 assay. As shown in Figure 1A, KSP, similar to CTX and Aidi, concentration-dependently reduced cell viabilities of MFCs. These data indicate that KSP may inhibit gastric cancer growth.

### KSP increases cell autophagy in cultured MFCs

Autophagy is involved in the biological process of gastric cancer [8]. Thus, we determined the effects of KSP on autophagy in MFCs by MDC staining and IFC analysis. As shown in Figure 1B-1D, KSP, as well as CTX and Aidi, increased the formation of autophagosome and the levels of LC3-II protein in MFCs, suggesting that KSP activates autophagy in gastric cancer cells.

### KSP induces the formations of mitophagosome and mitolysosome in MFCs

**Figure 2:**
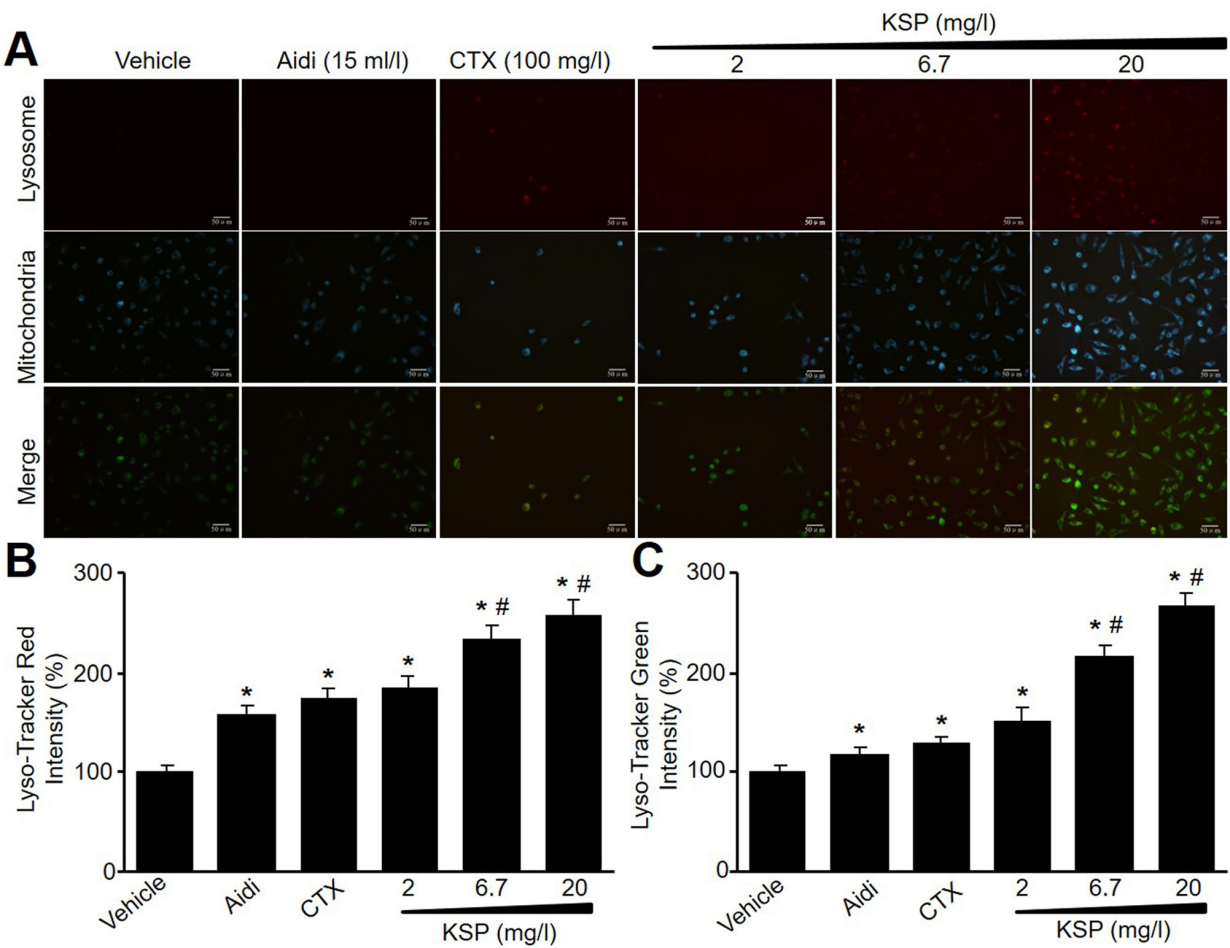
KSP induces the formations of mitophagosome and mitolysosome in MFCs MFCs were incubated with Aidi (15 ml/l), CTX (100 mg/l), and KSP (2, 6.7, 20 mg/l) for 48 hours. (**A**) Mitochondria and lysosome were detected by Lyso-Tracker Red and Mito-Tracker Green. The presented picture is a representative picture from three independent experiments. (**B** and **C**) The quantitative analyses of lysosomal in B and mitochondria in C were performed. N is 3 in each group. *P < 0.05 *VS* vehicle, ^#^*P* < 0.05 *VS* 2 mg/ml KSP.

Both mitochondria and lysosome are critical to the formation of autophagosome in cells [9, 10]. We next detected mitophagosome and mitolysosome by Mito-Tracker Green and Lyso-Tracker Red. As indicated in Figure 2A-2C, KSP, CTX, and Aidi increased the fluorescence intensities of Mito-Tracker Green and Lyso-Tracker Red in MFCs, further supporting that KSP increases cell autophagy in gastric cancer cells.

### KSP suppresses the *in situ* growth of gastric cancer in mice

**Figure 3:**
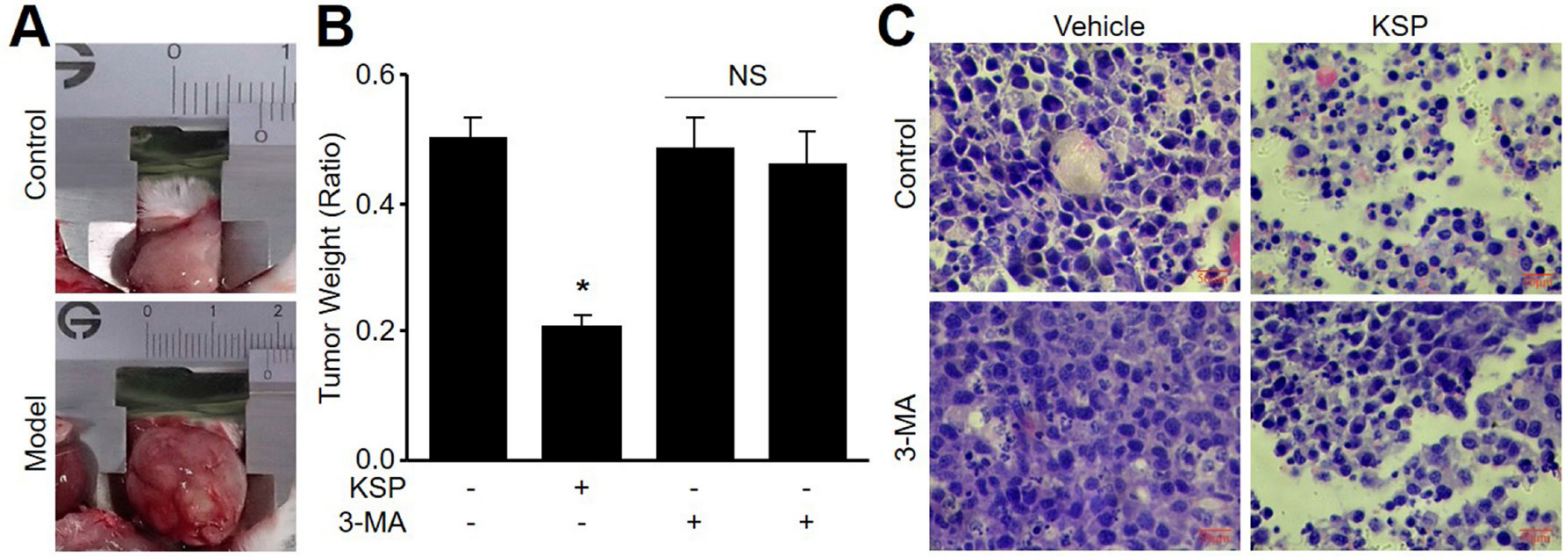
KSP suppresses the growth of gastric cancer in mice Nude mice received MFCs transplantations and then were treated with KSP (20 ml/kg/day) in presence or absence of 3-MA (10 mg/kg/day) for two weeks. (**A**) Morphology of gastric cancer in mice. (**B**) Tumor weight of gastric cancer in mice. (**C**) HE staining of gastric cancer in mice. 5–10 mice in each group. *P < 0.05 VS Control. NS indicates no significance.

To evaluate the effects of KSP on tumor growth *in vivo*, we generated the xenograft model of mouse gastric cancer by injecting MFCs into nude mouse subcutaneously. As shown in Figure 3A, transplantation of MFCs into nude mice induced the *in situ* formation of gastric cancer. Two-week treatments of KSP dramatically reduced the tumor weight (Figure 3B). HE staining indicated that KSP reduced the cell numbers of MFCs in tumor tissues (Figure 3C). These data demonstrate that KSP is considered as an anti-cancer drug on gastric cancer.

### KSP via induction of autophagy inhibits gastric cancer growth *in vivo*

**Figure 4:**
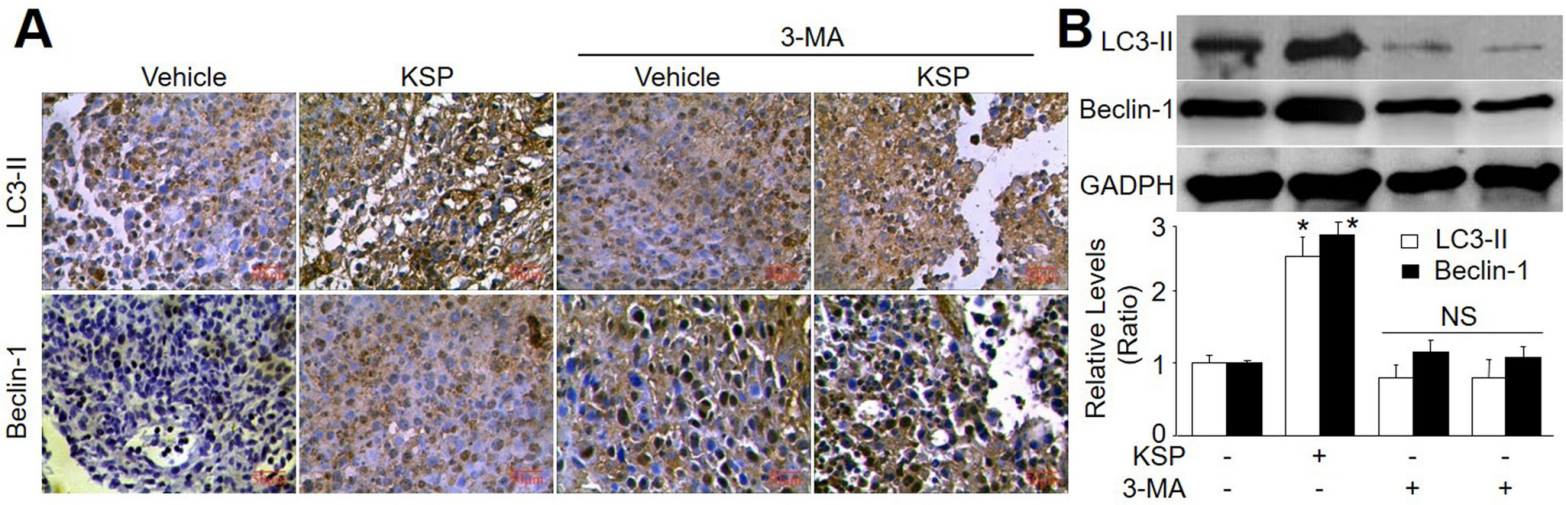
KSP increases autophagy of gastric cancer in mice Nude mice received MFCs transplantations and then were treated with KSP (20 ml/kg/day) in presence or absence of 3-MA (10 mg/kg/day) for two weeks. (**A**) IHC analyses of LC3-II and beclin-1 were performed in tissues of gastric cancer from mice. (**B**) The levels of LC3-II and beclin-1 were determined in tumor tissues of gastric cancer in mice by Western blot. 5–10 mice in each group. *P < 0.05 *VS* Control. NS indicates no significance.

To determine the role of autophagy in KSP-reduced gastric cancer growth in mice, 3-MA was administrated into nude mice to block autophagy [11]. As depicted in Figure 3B–3C, co-treatment of 3-MA abolished the inhibitory effects of KSP on gastric cancer growth including tumor weight and cell numbers. The role of autophagy in KSP-inhibited gastric cancer growth was further confirmed by assaying the protein levels of LC3-II and beclin-1 in tumor tissue. As shown in Figure 4A–4B, KSP significantly increased the levels of LC3-II and beclin-1 in tumor tissues, which was ablated by 3-MA, suggesting that KSP via induction of autophagy inhibits gastric cancer growth *in vivo.*

### KSP increases cathepsin L in gastric cancer *in vivo*

**Figure 5:**
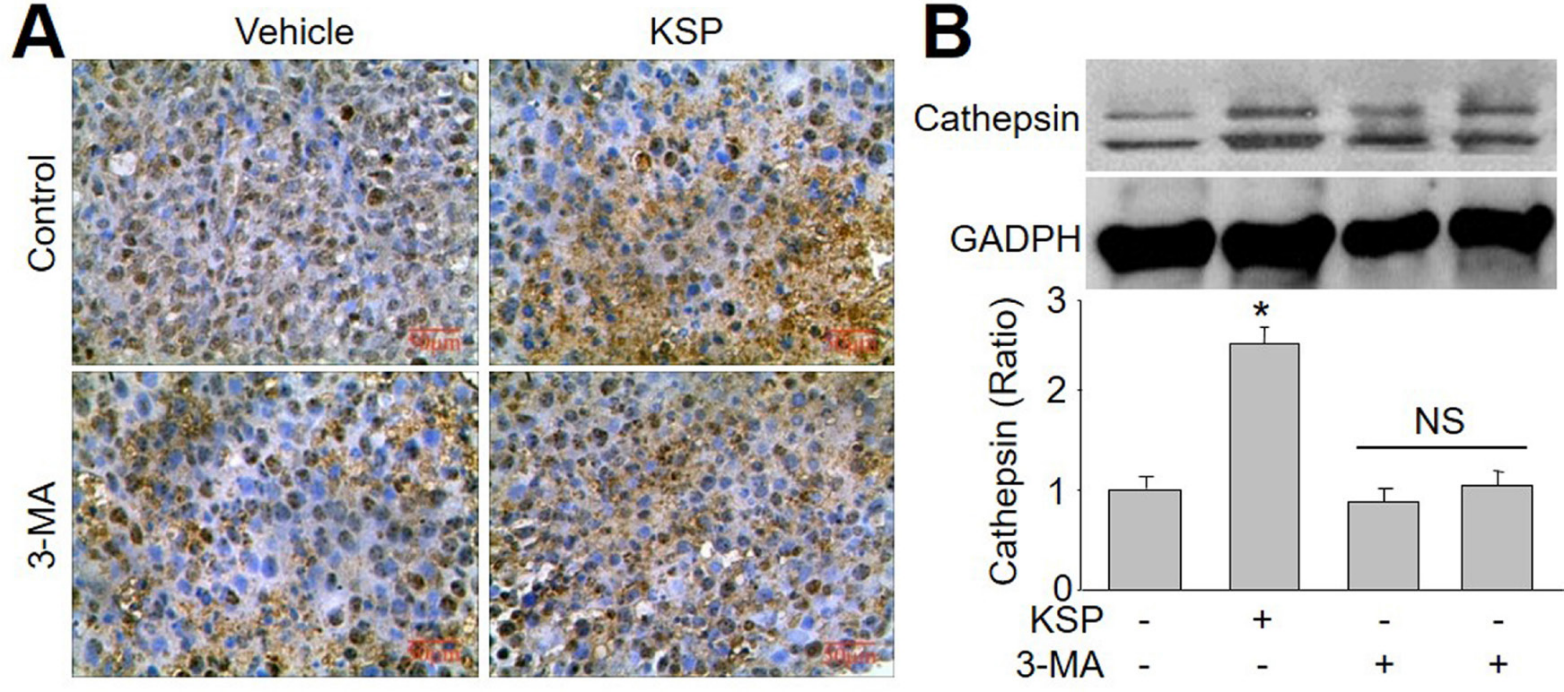
KSP increases the switch from autophagy to apoptotic death of gastric cancer in mice Nude mice received MFCs transplantations and then were treated with KSP (20 ml/kg/day) in presence or absence of 3-MA (10 mg/kg/day) for two weeks. (**A**) IHC analysis of cathepsin L was performed in tumor of gastric cancer in mice. (**B**) The levels of cathepsin L was determined in tumor tissues of gastric cancer in mice by Western blot. 5–10 mice in each group. *P < 0.05 *VS* Control. NS indicates no significance.

Cathepsin L plays a key in the switch from autophagy to apoptosis [12, 13]. Thus, we measured the levels of cathepsin L in tumor tissue by IHC analysis (Figure 5A) and Western blot analysis (Figure 5B). The expressional level of cathepsin L was enhanced by KSP. However, co-administration of 3-MA abrogated the effects of KSP on the upregulation of cathepsin L protein in tumor tissue.

### KSP induces apoptosis of gastric cancer in mice

**Figure 6:**
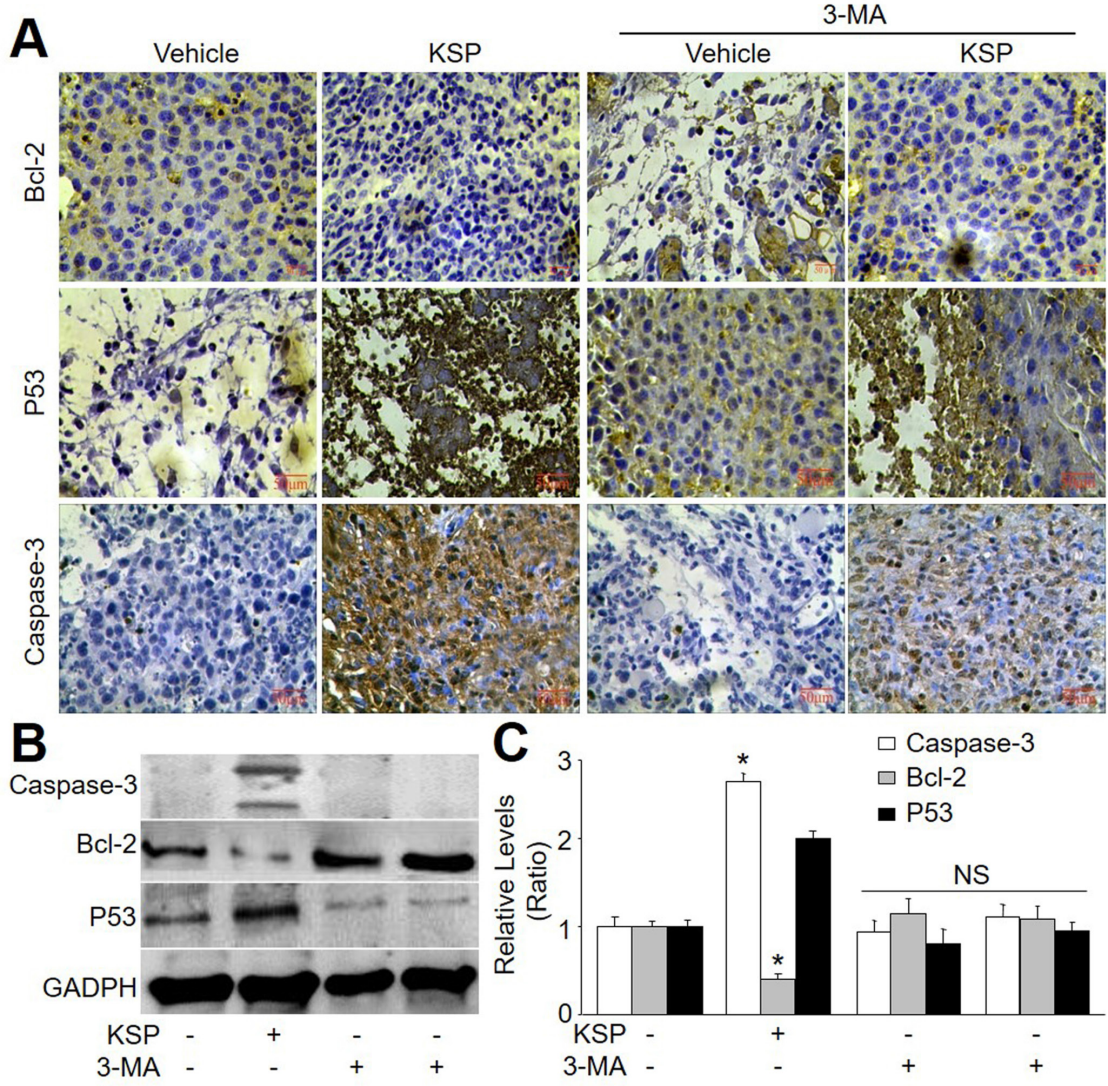
KSP induces cell apoptosis of gastric cancer in mice Nude mice were received MFCs transplantations and then were treated with KSP (20 ml/kg/day) in presence or absence of 3-MA (10 mg/kg/day) for two weeks. (**A**) IHC analyses of bcl-2, p53, and caspase-3 were performed in tumor of gastric cancer in mice. (**B**) The levels of bcl-2, p53, and caspase-3 were determined in tumor tissues of gastric cancer in mice by Western blot. (**C**) Quantitative analysis of results from B. 5–10 mice in each group. *P < 0.05 *VS* Control. NS indicates no significance.

Emerging evidence suggests that cross-talk occurs between autophagic and apoptotic pathways [14]. Thus, we also examined the role of apoptosis in KSP-inhibited gastric cancer growth. The apoptosis-related proteins including p53, bcl-2, and caspase-3 were assayed by analyses of IHC and western blot. As indicated in Figure 6A–6C, KSP increased the apoptotic protein levels of p53 and caspase-3, but decreased the level of bcl-2, which functions as anti-apoptosis [15]. These effects of KSP on apoptosis-related proteins were attenuated by 3-MA, indicating that KSP-induced apoptosis of gastric cancer is dependent on autophagy.

## DISCUSSION

Gastric cancer is the third leading cause of cancer death in the world [16, 17]. Although the survival rate of gastric cancer patients is improved with the development of chemotherapy in the past years, the long-term survival rates of these patients are still not satisfied. In this study, we developed a new prescription named after KSP and observed that KSP inhibits tumor growth in the xenograft model of mouse gastric cancer.

Mechanically, the anti-cancer effects of KSP is mediated by induction of autophagy in gastric cancer cells. In the process of autophagy, a phagophore from endoplasmic reticulum or plasma membrane forms autophagosomes, which depend on beclin-2, LC3-II, etc [18]. In this study, we observed that KSP promoted the formation of autophagosomes, mitophagosome and mitolysosome, and increased beclin-2 and LC3-II protein levels. This raised subsequent reduction of cell survival by destroying the cytosol and organelles, leading to cell death. KSP functions as an autophagy inducer in gastric cancer cells. Actually, some papers have reported that traditional Chinese medicine is able to regulate autophagy [19, 20], supporting our conclusion.

In summary, we have developed a novel traditional Chinese medicine of KSP to prevent the growth of gastric cancer in mice. The underlying mechanism is related to the induction of autophagy of gastric cancer cells. Our results provided another choice to prevent and treat patients with gastric cancer or other gastrointestinal tumors clinically.

## MATERIALS AND METHODS

### Animals and Materials

KSP is a Chinese medicinal formulation that is available in capsule form. The formula contains lucid ganoderma, silymarin, resveratrol, hedyotis diffusa, epimedium, oysters, and codonopsis pilosula. KSP crude drugs were purchased from Tong-Ren-Tang Company (Beijing, China). Aidi injection was purchased from YiBai Pharmaceutical Company (Guizhou, China). CCK8 kit was obtained from Dojindo Laboratories (Kumamoto, Japan). Lyso-Tracker Red C1046 and Mito-Tracker Green C1048 were brought from Beyotime Biotechnology (Shanghai, China). Primary antibodies against bcl-2 (ab59348), p53 (ab31333), capase-3 (ab2302), beclin-1 (ab62557), LC3-II (ab48394), and cathepsin L (ab6314) were purchased from Abcam (United States). All female BAL b/c nude mice were purchased from Institute of Laboratory Animal Sciences, Cams&Pumc; Beijing, China.

### Cell culture and analysis for cell viability

The mouse gastric cancer cell line (MFC) was purchased from the Cell Bank of the Chinese Academy of Medical Sciences, Beijing, China. Cell viability was measured by CCK kit according to the manufacture's instruction as described previously [21].

### MDC staining of autophagic vacuoles

MDC staining of autophagic vacuoles was performed for autophagy analysis as previously described [22].

### Establishment of the xenograft model of gastric cancer in mice

The xenograft model of gastric cancer was established as described previously [23].). Following two days of growth, animals were randomly divided into 4 groups. Group 1: Vehicle group; Group 2: KSP group; Group 3: 3-MA group; Group 4: KSP plus 3-MA group. Mice in group 1 intragastrically administrated with saline for 2 weeks. Mice in group 2 received gavage with KSP (20 ml/kg/day) for 2 weeks. Mice in group 3 received treatment of 3-MA (10 mg/kg/day) in drinking water for 2 weeks. Mice in group 4 received KSP plus 3-MA for 2 weeks. This study was carried out in strictly accordance with the recommendations in the Guide for the Care and Use of Laboratory Animals of the National Institutes of Health. The protocol was approved by the Committee on the Ethics of Animal Experiments of Xinxiang Medical University.

### Immunohistochemistry (IHC)

As described previously [24], the tissue was fixed in 4% paraformaldehyde overnight, and then processed, embedded in paraffin, and sectioned at 4 μm. Semiquantitative analysis of tissue immunoreactivity was done as described previously with modifications [25].

### Western blot analysis

As described previously [26, 27], total proteins were subjected to Western blots using ECL-Plus.

### Statistical analysis

Values are expressed as mean ± SEM. Statistical comparisons were performed by using one-way ANOVA. Intergroup differences were analyzed using Bonferroni's pos*t*-test. Analysis of dose-course studies was performed with repeated measures ANOVA. *P* values less than 0.05 were considered as significant.
